# Verbal fluency difficulties in aphasia: A combination of lexical and executive control deficits

**DOI:** 10.1111/1460-6984.12710

**Published:** 2022-03-23

**Authors:** Arpita Bose, Abhijeet Patra, Georgia Eleftheria Antoniou, Rachael C. Stickland, Eva Belke

**Affiliations:** ^1^ School of Psychology and Clinical Language Sciences University of Reading Reading UK; ^2^ Manchester Metropolitan University Manchester UK; ^3^ Physical Therapy and Human Movement Sciences, Feinberg School of Medicine Northwestern University Evanston IL USA; ^4^ Ruhr‐Universität Bochum Bochum Germany

**Keywords:** aphasia, clusters, executive control, letter fluency, semantic fluency, switches, timing

## Abstract

**Background:**

Verbal fluency tasks are routinely used in clinical assessment and research studies of aphasia. People with aphasia produce fewer items in verbal fluency tasks. It remains unclear if their output is limited solely by their lexical difficulties and/or has a basis in their executive control abilities. Recent research has illustrated that detailed characterization of verbal fluency performance using temporal characteristics of words retrieved, clustering and switching, and pause durations, along with separate measures of executive control stands to inform our understanding of the lexical and cognitive underpinnings of verbal fluency in aphasia.

**Aims:**

To determine the locus of the verbal fluency difficulties in aphasia, we compared semantic and letter fluency trials between people with aphasia and healthy control participants using a wide range of variables to capture the performance between the two groups. The groups were also tested on separate measures of executive control to determine the relationship amongst these tasks and fluency performance.

**Methods & Procedures:**

Semantic (animal) and letter (F, A, S) fluency data for 60s trials were collected from 14 people with aphasia (PWA) and 24 healthy adult controls (HC). Variables, such as number of correct responses, clustering and switching analyses, were performed along with temporal measures of the retrieved words (response latencies) and pause durations. Participants performed executive control tasks to measure inhibitory control, mental‐set shifting and memory span.

**Outcomes & Results:**

Compared with HC, PWA produced fewer correct responses, showed greater difficulty with the letter fluency condition, were slower in getting started with the trials, showed slower retrieval times as noted in within‐ and between‐cluster pause durations, and switched less often. Despite these retrieval difficulties, PWA showed a similar decline in the rate of recall to HC, and had similar cluster size. Executive control measures correlated primarily with the letter fluency variables: mostly for PWA and in one instance for HC.

**Conclusions & Implications:**

Poorer performance for PWA is a combination of difficulties in both the lexical and executive components of the verbal fluency task. Our findings highlight the importance of detailed characterization of fluency performance in deciphering the underlying mechanism of retrieval difficulties in aphasia, and illustrate the importance of using letter fluency trials to tap into executive control processes.

**WHAT THIS PAPER ADDS:**

**What is already known on the subject**

- PWA typically show impaired performance in verbal fluency tasks. It is debated whether this impaired performance is a result of their lexical difficulties or executive control difficulties, or a combination of both. This debate continues because previous studies have mostly used semantic fluency condition without including letter fluency condition; used a limited range of variables (e.g., number of correct responses); and not included separate executive control measures to explain the performance pattern in aphasia. This research addresses these outstanding issues to determine the specific contribution of lexical and executive control processes in verbal fluency in aphasia by including: both semantic and letter fluency conditions; a wide range of variables to identify the relative contribution of lexical and executive control mechanisms; and independent measures of executive control.

**What this paper adds to existing knowledge**

- Using the multidimensional analysis approach for verbal fluency performance from both semantic and letter fluency conditions, this is the first study to systematically demonstrate that PWA had difficulties in both lexical *and* executive control components of the task. At the individual level, PWA had greater difficulty on the letter fluency condition compared with semantic fluency. We observed significant correlations between the executive control measures and verbal fluency measures primarily for the letter fluency condition. This research makes a significant contribution to our understanding of lexical and executive control aspects in word production in aphasia.

**What are the potential or actual clinical implications of this work?**

- From a clinical perspective, this research highlights the importance of using a full range of verbal fluency and executive control measures to tap into the lexical as well as executive control abilities of PWA, and also the utility of using letter fluency to tap into the executive control processes in PWA.

## INTRODUCTION

Verbal fluency or word‐generation tasks have been widely used in clinical practice and research studies in a range of populations. Typically, participants are required to generate as many unique words as possible within a fixed period of time, usually 60 s, in accordance with certain criteria. Most common types of criteria used are semantic or category (e.g., animal names, supermarket items) and letter or phonemic (e.g., words beginning with a specific letter or phoneme). Successful performance relies on the integrity of both lexical and executive control abilities (e.g., Bittner & Crowe, 2007; Patra et al., 2020a, 2020b; Shao et al., 2014; Troyer, 2000). Specifically, its success depends on the integrity of lexical and semantic stores; on different executive control processes, such as initiation of word retrieval, lexical search, systematic monitoring of verbal output, inhibition of previously named words and the appropriate speed of performance. The quick nature of administration and the lack of need for specialized equipment or training have proved it to be a widely used task to examine deficits in language and cognition in a variety of different neurological disorders (Thiele et al., 2016).

It is well established that people with aphasia (PWA) produce few exemplars during a verbal fluency task (Adams et al., 1989; Roberts & Dorze, 1994; Sarno et al., 2005; Baldo et al., 2010; Arroyo‐Anlló et al., 2012; Kiran et al., 2014; Bose et al., 2017; Faroqi‐Shah & Milman, 2018). Lexical difficulties have been proposed to be the main driver for the impaired performance in aphasia (e.g., Adams et al., 1989; Arroyo‐Anlló et al., 2012; Bose et al., 2017). However, the recent literature has also implicated difficulties with executive control as an underlying cause for the impaired performance (Bose et al., 2017; Faroqi‐Shah et al., 2018; Patra et al., 2020a). The debate continues as to whether limited output in aphasia is a result of lexical difficulties or executive control difficulties, or a combination of both.

Previous research has not been able to provide a definitive answer to this debate and has been limited by multiple factors. First, most studies have used only semantic fluency trials, whereas research has shown that a comparison of semantic versus letter fluency enables us to better tap into the contribution of executive control processes (Luo et al., 2010; Friesen et al., 2015; Patra et al., 2020a, 2020b). Second, the literature beyond aphasia has provided us with various analyses options ranging from the number of correct responses to more involved timing analyses (Thiele et al., 2016; Luo et al., 2010). These analyses have provided more definite answers to the debate of lexical versus executive mechanism control in verbal fluency performance in healthy bilingual adults (Luo et al., 2010; Patra et al., 2020b) and in bilinguals with aphasia (Patra et al., 2020a). Despite the availability of these analysis options from the literature, most studies in aphasia have limited themselves to the number of correct responses, clustering and switching measures, and only occasionally used timing measures. Third, recent studies that have used independent measures of executive control have been able to better inform the above debate (e.g., Shao et al., 2014; Patra et al., 2020a, 2020b). Table 1 provides a description of these variables and identifies whether these variables tap into lexical and/or executive control components. A comprehensive understanding of the mechanisms and processes underlying verbal fluency performance is of great importance in aphasia as it would elucidate the underlying nature of the word retrieval deficits in aphasia. This in turn stands to improve assessment and rehabilitation for neurological conditions.

**TABLE 1:** Description of the verbal fluency variables and relative contribution of lexical and executive control processes for each of these variables

**Parameters**	**Description**	**Lexical processes**	**Executive control processes**
*Quantitative analysis*			
Number of correct responses (CR)	Number of words generated in 60 s excluding errors. Measures word‐retrieval abilities	√	√
Fluency difference score (FDS) ^a^	Difference in the number of correct responses between semantic and letter fluency conditions as a proportion of correct responses in the semantic condition. Measures the ability to maintain the performance in the demanding condition (i.e., letter fluency)		√
*Time‐course analysis* ^b,c^			
1st‐RT	Duration from the beginning of the trial to the onset of first response. Measures the preparation time	√	
Sub‐RT	Average of time intervals from the onset of first response to the onset of each subsequent response. Estimate for mean retrieval latency and represents the time point at which half of the total responses have been generated. Mean latency is not equivalent to retrieval speed; instead, it mirrors the slope of the decline in lexical retrieval over the course of a trial		√
*Clustering and switching analysis* *^d^*			
Cluster size	Strategic process that helps to generate words within a subcategory and uses the speaker's ability to access words within subcategories	√	
Number of switches	Strategic process to shift efficiently to a new subcategory when a subcategory is exhausted		√
Within‐cluster pauses	Mean time differences between each successive word within the same cluster	√	
Between‐cluster pauses	Mean time difference between the onset time of the last word of a cluster and first word of the consecutive cluster		√

*Sources*: Adapted from Patra et al. (2020a, 2020b). ^a^Friesen et al. (2015); ^b^Luo et al. (2010); ^c^Sandoval et al. (2010); and ^d^Troyer et al. (1997).

In this paper we set out to resolve and better inform the debate of lexical versus executive control difficulties in verbal fluency performance in aphasia. We tackled this question in two ways. First, we provided a detailed characterization of verbal fluency performance to determine the components that were impaired. The analysis possibilities—ranging from number of correct, clustering, switching to more detailed analyses such as retrieval times of words and time‐course analysis—afforded us the opportunity to differentially tap into the various components of lexical and executive control contribution. For example, the ability to switch between subcategories and the duration of between‐cluster pauses have been attributed to the executive component of the task (Troyer et al., 1997; Rosen et al., 2005; Bose et al., 2017); cluster size and duration of within‐cluster pauses have been attributed to the lexical component of the task (e.g., Tröster et al., 1998; Raboutet et al., 2010). Similarly, a newly derived variable, fluency difference score (FDS), has been suggested to capture the role of executive control in a fluency task (Friesen et al., 2015). As noted in Table 1, we used multiple variables that have been used across the literature to capture the contribution of lexical and executive control components of the verbal fluency task. Thus, if the underlying difficulties in aphasia are predominantly with the lexical component, then differences between aphasia and control speakers will be most prominent on variables that tap into the lexical components (e.g., cluster size, within‐cluster pauses, first‐response time—1st‐RT). In contrast, if the underlying difficulties in aphasia are with the executive components of the verbal fluency task, then most differences between aphasia and control speakers will be on the variables that tap into the executive control component of the task (e.g., FDS, number of switches, Sub‐RT, between‐cluster pause).

Second, to establish the executive control underpinnings of the verbal fluency performance, we collected several independent measures of executive control processes, namely the Stroop task (measuring selective inhibition; Scott & Wilshire, 2010), the Trail Making Test (TMT; measuring shifting between mental sets; Reitan, 1986), and the auditory backward digit span (measuring working memory; Wechsler, 1997). This two‐pronged approach (detailed characterization of verbal fluency to determine the components of lexical or executive control, along with independent measures of executive control) enabled us to specify the relative lexical and executive control involvement in verbal fluency performance in aphasia.

As mentioned, PWA produce fewer exemplars than control participants, often along with smaller cluster sizes and fewer number of switches (e.g., Adams et al., 1989; Roberts & Le Dorze, 1997; Baldo et al., 2010; Arroyo‐Anlló et al., 2012; Kiran et al., 2014; Bose et al., 2017; Faroqi‐Shah & Milman, 2018). The productivity reduces as a function of time (Adams et al., 1989; Bose et al., 2017) and the possibility remains that performance might be influenced by executive control impairment (Bose et al., 2017; Carpenter et al., 2020; Faroqi‐Shah et al., 2018; Patra et al., 2020a). Using semantic fluency data from a large cohort of PWA and control speakers, Bose et al. (2017) demonstrated that PWA produced significantly fewer number of words, smaller cluster sizes, longer within‐ and between‐cluster pauses, and that the decrease in number of switches significantly correlated with an increase in between‐cluster pauses. Bose et al. concluded that the poorer performance of PWA arose primarily from a lexical retrieval deficit but speculated that executive control deficits may have also contributed to the performance deficits. Their conclusion was based on the observations that variables that depend heavily on executive control components, such as switching and between‐cluster pauses, showed significant differences between PWA and control participants. However, their study did not include independent measures of executive control nor did they have letter fluency trials. Several authors have put forward the argument that a letter fluency task better taps into executive control processes; a greater emphasis is placed on linguistic abilities in a semantic fluency task (Delis et al., 2001; Luo et al., 2010; Paap et al., 2017; Patra et al., 2020a; Sandoval et al., 2010; Shao et al., 2014; see Thiele et al., 2016, for a review on this issue; along with Gordon et al., 2018, and Whiteside et al., 2016, for a contrasting view). It is primarily because semantic fluency taps on to the existing semantic links and organization of our mental lexicon, for instance, when coming up with names of grocery items for a shopping list. However, in the letter fluency condition, participants are required to produce words starting with a letter or phoneme, which is not commonly practiced in our everyday life. Successful performance in the letter fluency condition requires the participant to come up with strategies and to inhibit the activation of related semantic concepts (e.g., Friesen et al., 2015; Luo et al., 2010).

Although in the monolingual aphasia literature, there exists no direct investigation of lexical and executive control contributions of verbal fluency along with independent measures of executive control measures, significant progress has been made in research studies amongst various bilingual healthy populations and also in bilingual aphasia (Carpeneter et al., 2020; Luo et al., 2010; Patra et al., 2020a, 2020b; Faroqi‐Shah et al., 2018). Faroqi‐Shah et al. (2018) investigated the relationship between semantic fluency and executive control (measured by the Stroop Colour Word Test; Golden & Freshwater, 1978) in three groups of PWA—English monolinguals, English‐dominant bilinguals and Tamil–English bilinguals—and three groups of healthy controls. The authors found that PWA produced fewer number of correct responses and demonstrated higher Stroop ratio (indicative of poorer executive control). Critically, the authors did not find any relationship between number of correct responses and the Stroop performance. The authors attribute the lack of a correlation between inhibitory control and semantic fluency to impaired cognitive control abilities in aphasia which were no longer available to support word retrieval. However, it is also possible that the Stroop task might not be an appropriate task to tap into the inhibitory control mechanisms relevant to semantic fluency. Alternatively, the lack of a letter fluency trial in their study meant it was not possible to reveal any relationship between executive control and other verbal fluency variables. It has been shown in previous studies that letter fluency trials stretch the executive control system much more than semantic trials do (Baldo et al., 2010; Friesen et al., 2015; Luo et al., 2010; Thiele et al., 2016; Troyer et al., 1997).

Involvement of executive control in verbal fluency performance has been established in two recent studies involving bilinguals with aphasia (Carpenter et al., 2020; Patra et al., 2020a). Carpenter et al (2020) manipulated the executive control demand in a verbal fluency task by asking participants to either respond in any language (self‐switch condition) or switch from one language to another within the task (forced switch). They found that bilinguals with aphasia had greater difficulty in the forced‐switch condition (greater demand on the executive control mechanism) but performed similarly to controls on the easier self‐switch condition. The authors concluded that bilinguals with aphasia were sensitive to the greater executive control requirement in the verbal fluency task. A more relevant study to the present study is that by Patra et al. (2020a), who examined the involvement of executive control processes in verbal fluency performance in a group of Bengali–English bilinguals with aphasia by including both semantic and letter fluency conditions as well as independent measures of executive control. The authors found bilinguals with aphasia performed poorly on the verbal fluency measures, where executive control demands were higher (e.g., letter fluency, FDS, number of switches and between‐cluster pauses). Moreover, these findings are consistent with the correlational analysis demonstrating a significant relationship between Stroop ratio and backward digit span) and verbal fluency variables (i.e., number of correct, 1st‐RT and number of switches) (Table 1).

## THE CURRENT INVESTIGATION, RESEARCH QUESTIONS AND PREDICTIONS

Despite progress made through the above‐mentioned studies, methodological and procedural differences are important determiners for the impact of findings and several issues remain unresolved. We strive to address these unresolved issues (discussed below) and seek to fill the gaps to determine the relative contribution of lexical and executive control processes during verbal fluency performance in aphasia.

- The above‐mentioned studies in aphasia, with the exception of Faroqi‐Shah and Milman (2018), have mostly investigated semantic fluency, whereas letter fluency remains underexplored in monolingual aphasia population. Our understanding of verbal fluency in monolingual aphasia will benefit from a systematic comparison of semantic and letter fluency. It would enable us to calculate variables, such as FDS, which has been suggested to capture the role of executive control in a fluency task. Individuals who can maintain better performance in the difficult letter fluency condition would show a smaller FDS score (for the calculation of FDS, see Table 1), which would be indicative of better executive control abilities (Friesen et al., 2015).
- Most studies on verbal fluency in aphasia have restricted their analysis only to the number of correct responses with few studies venturing into clustering and switching analyses. As the verbal fluency task places a premium on rapid search and retrieval, temporal measures of performance (i.e., timing for the retrieved words, clustering and switching) and information processing speeds (i.e., time interval required to produce each word as a function of its position in the sequence) provide insights into the linguistic and executive control strategies (e.g., Crowe, 1998; Luo et al., 2010; Sandoval et al., 2010; Patra et al., 2020a, 2020b). There have been some attempts to obtain timing measures for verbal fluency in aphasia (Adams et al., 1989; Bose et al., 2017), but not as extensively as in other literature (e.g., healthy bilingual versus monolingual literature: Luo et al., 2010; Sandoval et al., 2010; Patra et al., 2020b; and bilinguals with aphasia: Patra et al., 2020a).

In the time‐course analysis, the number of words generated over the 60 s time interval is grouped into 5 s time bins, with declining response rate presented by plotting the number of words produced as a function of time. The declining rate of recall is reflected by the slope of the resulting function; the declining curve is exponential (Wixted & Rohrer, 1994). Two parameters were generated from this graph: first‐response time (1st‐RT) and subsequent‐response time (Sub‐RT) (see Table 1 for the definition of these measures; and the methods section below). The most salient (and often ignored) measure derived from this analysis is the Sub‐RT (also referred to as the mean retrieval latency), which corresponds to the midpoint of the exponentially declining curve of retrieval. This measure has the potential to characterize the organization of the mental lexicon as it taps on to the declining rate of verbal recall rather than overall speed of retrieval (Rohrer et al., 1995). Such a detailed endeavour has not yet been attempted with monolingual aphasia; the present research will be a step change in our understanding of word production and executive control in aphasia.

- There is a lack of research addressing the relationship between verbal fluency and independent measures of executive control in aphasia. To the best of our knowledge there only two studies (Faroqi‐Shah et al., 2018; Patra et al., 2020a) have attempted to link verbal fluency performance with separate executive control measures in bilinguals with aphasia. However, Faroqi‐Shah et al. measured only the number of correct responses for the semantic fluency task and inhibitory control for the executive control measure. In Patra et al.’s study, correlations were conducted between independent executive control measures (inhibitory control, mental‐set shifting measures and working memory) and a range of verbal fluency measures. However, correlations were not conducted separately for the semantic and letter fluency conditions due to small sample size. Investigating the contribution of executive control separately for semantic and letter fluency conditions would contribute to the debate of whether letter fluency places greater demands on the executive control mechanisms, compared with semantic fluency. Therefore, in the present study we examined three independent measures of executive control abilities—inhibitory control (verbal Stroop Test), mental set‐shifting (Trail Making Test) and working memory (backward digit span)—based on Miyake et al.’s (2000) influential executive control framework. Importantly, we conducted the correlation separately between independent executive control measures and two verbal fluency conditions (semantic and letter). The choice of these tasks (i.e., verbal Stroop, TMT and backward digit span) was primarily based on the feasibility of using them with the neurological populations (e.g., aphasia) and availability of literature on these tasks for comparisons.

To summarize, we address the significant gaps in aphasia and verbal fluency research by including both semantic and letter fluency trials; implementing extensive characterization of verbal fluency performance along with independent measures of executive control. Specifically, we compared verbal fluency performance between 14 English monolingual PWA and 24 healthy control adults (HC). We collected semantic (animal) and letter (F, A, S) fluency data for 60 s. We quantified the participants’ performance in a range of variables (see Table 1 for a complete description of these variables) and analysed the data at both the group and individual levels. Given that heterogeneity in performance is a hallmark feature of any aphasia group study, we provide individual level analyses for the fluency variables and executive control measures. This was undertaken to provide in‐depth understanding of the performance by participants with aphasia, which is often missed if we rely solely on group means.

The following are the specific research aims and predictions:

- To determine differences in verbal fluency performance across semantic and letter fluency (quantitative, time course, as well as clustering and switching analysis) between PWA and HC.

We predict that if a deficit in PWA is primarily in the lexical component of the task, then we would see differences between the groups specifically on variables that tap into the lexical component (e.g., 1st‐RT, cluster size and within‐cluster pause). In contrast, if PWAs’ deficit is in the executive component of the task, then we will see prominent differences between the groups on the variables that tap into the executive control component (e.g., FDS, number of switches and slope). Based on the literature, we anticipate that at the group level PWA will show differences in a range of variables that taps into both the lexical and executive control components of the task.

- To establish the relationship between verbal fluency performance and executive control abilities for PWA and HC.

Based on previous research we expect that executive control measures (especially, inhibitory control and mental‐set shifting) may correlate significantly with verbal fluency variables for both PWA and HC (Patra et al., 2020a), but the strength of these correlations might be different in the two groups. Specifically, under this prediction we expect PWA to have a stronger correlation between executive control and verbal fluency measures compared with HC. Additionally, if letter fluency demands greater executive control, we would expect correlations to be stronger between the executive control measures and letter fluency condition, compared with semantic fluency condition.

## METHODS

### Participants and background test battery

We recruited 14 PWA (seven male, seven female) and 24 age‐ and education‐matched HC (10 male, 14 female). There were no significant differences between the groups with regard to age (PWA, mean = 63.3 years, SD = 9.4; HC, mean = 67.3 years, SD = 10.1; *t* = 1.03; *p* = 0.29) and level of education (PWA, mean = 13.8 years, SD = 2.4; HC, mean = 14.0 years, SD = 3.06; *t* = 0.86; *p* = 0.88). All participants were monolingual speakers of British English, right‐handed (PWA pre‐morbidly right‐handed) and had at least 10 years of education. Inclusion criteria for PWA were: a single left hemisphere cardiovascular accident as determined by neuroradiological and/or neurological examinations; a diagnosis of aphasia on standardized clinical tests (Boston Diagnostic Aphasia Examination—BDAE; Goodglass et al., 2001); at least 12 months post‐stroke; no history of other neurological illness, psychiatric disorders or substance abuse; no visual field or sensory perceptual deficits; and no other significant cognitive deficits. All procedures were approved by the Institutional Research Ethics Committee (ethical approval code 2012/049AB).

The BDAE‐short form (Goodglass et al., 2001) was administered to determine the type and severity of aphasia. Appendix A presents demographic information, aphasia type, severity and scores on various linguistic measures. The group included five individuals with Broca's aphasia, one with transcortical motor aphasia, one with mixed aphasia, four with anomic aphasia, and three with conduction aphasia. BDAE aphasia severity ratings ranged from 1 to 4 (mean = 3.2, SD = 0.9; with 1 as the most severe and 5 the least severe). In addition, a comprehensive evaluation of participants’ single word comprehension and production was performed using subtests from the Psycholinguistic Assessments of Language Processing in Aphasia (PALPA; Kay et al., 1992); the three‐picture version of the Pyramids and Palm Trees Test (PPT; Howard & Patterson, 1992) and the Philadelphia Naming Test (PNT; Roach et al., 1996). Using various tasks, this battery measured input phonological abilities (i.e., PALPA subtests 2 and 4), output phonological abilities (PALPA subtests 9 and 8), conceptual semantic processing (PPT‐3 picture version), lexico‐semantic processing (PALPA subtest 47 and Philadelphia Lexical Comprehension task), and naming abilities (PNT). As a group they showed variable impairments both for input and output phonology; better preserved conceptual and lexical semantics (PPT scores ranged from 90% to 98%, mean = 96%, SD = 2.4); a wide range in picture naming abilities (PNT scores ranged from 34% to 96%, mean = 76.9%, SD = 17.2).

### Verbal fluency measures

#### Trials and procedure

Participants completed two verbal fluency conditions—semantic (animal) and letter (F, A, S). For the animal fluency task, participants were asked to name as many animals they could in 60 s. To ensure spontaneous cognitive and search strategies, no guidelines were provided regarding how the participants should generate and organize their production. For the letter fluency task, participants were asked to generate words beginning with the letters, F, A and S, each for a 60 s duration. The restrictions for the letter trials were to produce unique words that are not proper nouns nor numbers (e.g., Singapore, seven), and to not produce variants of the same words (e.g., shop, shopper, shopping). The order of the fluency conditions was randomized across participants; however, the trials were blocked by condition. Each participant was tested individually in a quiet room. After providing the instruction, the participant started a trial only when the tester said ‘start’. This ensured that there was a definitive starting point for each trial. Responses were recorded with a digital voice recorder and all responses (including repetition and errors) were transcribed verbatim.

#### Data coding and analysis

Each naming response was time‐stamped using PRAAT (Boersma & Weenink, 2015). The time‐stamping enabled us to index the onset of a response from the onset of the trial (i.e., ‘start’), which allowed us to calculate the variables in time‐course analysis. We measured the following variables for each trial.

#### Number of correct responses (CR)

Number of responses produced in 1 min excluding any errors. In semantic fluency, errors were repetitions, non‐words, non‐animal names and unintelligible words. In letter fluency, errors were repetition, non‐words, unintelligible words, words beginning with a different letter (e.g., *sun* as a response for letter A), and proper nouns (e.g., *America* as a response for letter A). Same word with different inflectional or derivational suffixes (e.g., swim, swimmer) were counted as single CR.

#### Fluency difference score (FDS)

This was the difference in the number of correct responses between semantic and letter fluency conditions as a proportion of correct responses in the semantic fluency condition (Friesen et al., 2015):

$$\begin{array}{ccc} FDS & = & \left(CR_{semantic\;fluency}-CR_{letter\;fluency}\right)\\ & & /CR_{semantic\;fluency} \end{array}$$

#### Time‐course analysis

We computed two variables (1st‐RT, Sub‐RT) based on the timing of the responses (Luo et al., 2010).

##### First‐RT (1st‐RT)

1st‐RT is the time interval from the beginning of the trial to the onset of the first response. The first response has been linked to the task preparation (Rohrer et al., 1995).

##### Subsequent‐RT (Sub‐RT)

Sub‐RT is defined as the mean value of retrieval latencies of each recalled item relative to the onset of recall. It is important to note that it is not equivalent to retrieval speed; it is best described as the declining rate of recall (Rohrer et al., 1995). Thus, Sub‐RT provides a good estimate for mean retrieval latency and represents the time point at which half of the total responses have been generated (Sandoval et al., 2010). A longer mean Sub‐RT indicates that performance extends later into the time course. In contrast, a shorter mean Sub‐RT would indicate a faster declining rate of retrieval because a large proportion of the responses were produced early during the trial, and this is often associated with structural loss to the mental lexicon (Rohrer et al., 1995).

#### Clustering and switching analyses

Four variables were calculated to characterize the clustering and switching abilities: cluster size, number of switches, within‐cluster pauses and between‐cluster pauses. Detailed procedure for clustering and switching analysis was based on Troyer (2000) and Troyer et al. (1997). Although repetition errors were excluded for CR calculations, they were retained for clustering and switching analysis as these are thought to be reflective of underlying cognitive processes regardless of whether they were included in total number of words generated (Troyer et al., 1997). The following four variables were generated after clustering the responses.

#### Cluster size

Cluster size was calculated beginning with the second word in each cluster. A cluster size of 0 was given for a single word (e.g., *cat*), a cluster size of 1 was given for two‐word clusters (e.g., *cat*, *dog* belong to pet animal cluster), and so on. Words that shared the same semantic subcategory constituted the semantic fluency cluster. In letter fluency, clustering was defined as successively generated words which fulfil any one of the following criteria (Troyer et al., 1997): words that begin with the same first two letters (*stop*, *stone*); words that differ only by a vowel sound regardless of the actual spelling (*sheep*, *ship*); words that rhyme (*stool*, *school*); or words that are homonyms (*son*, *sun*). Mean cluster size for a trial was calculated by adding the size of each cluster and dividing this total score by the number of clusters.

#### Number of switches

This refers to the number of transitions between clusters. For example, *dog*, *cat*; *snake*, *lizard*; *bear*; *horse*, *cow*, *goat* contain three switches—before snake, before bear and before horse. *Leopard*, *cheetah*; *kangaroo*, *koala bear*; *robin*, *sparrow*, *crow*; *chimpanzee*, *orang‐utan*, *baboon* have three switches—before kangaroo, robin and chimpanzee. Similarly, in letter fluency, *fragile*, *fraught*, *fray*; *fan*, *fat*; *fly*, *flower*, *flute* contain two switches before *fan* and before *fly*.

#### Within‐cluster pause

Within‐cluster pause refers to the mean time difference between successive words within a cluster. For example, a three‐word cluster *pig*, *cow*, *horse*, with onset times for *pig*, *cow* and *horse* was being 5, 7 and 8, respectively. Within‐cluster pause for this farm animal cluster will be ({(7 – 5) + (8 – 7)}/2 = 1.5 s). Mean within‐cluster pause for a trial was calculated by adding the values of the within‐cluster pauses for each of the clusters and dividing this total value by the number of clusters.

#### Between‐cluster pauses

Between‐cluster pauses refer to the time difference between the onset time of the last word of a cluster and first word of the consecutive cluster. Mean between‐cluster pause for a trial was calculated by adding the values of the between‐cluster pauses for transitions (i.e., switches) and dividing this total value by the number of switches.

#### Executive control tasks

Inhibitory control (Word Stroop Test). The adapted version of the computerized Stroop Task from Scott and Wilshire (2010) was used for this study. This task has been successfully used to measure inhibitory control in different populations (e.g., Patra et al., 2020a, 2020b). It consisted of six colours and their names: red, green, blue, yellow, orange and purple. The task was divided into two conditions, neutral and incongruent. In the neutral condition, participants named the colour of differently coloured rectangles as quickly and accurately as possible. A series of 50 coloured rectangles, each in one of the six colours, was presented in a random order, such that two successive trials never had the same colour. In the incongruent condition, participants were instructed to name the font colour of the coloured words as quickly and accurately as possible. A series of 50 coloured words were shown one at a time on the screen in a random order, each of which was presented in a colour other than the word's name (e.g., red in a green colour). The procedure was the same for both conditions. Each condition began with six practice trials. Both conditions were completed during a single session with the neutral condition first followed by the incongruent condition. The onset of each stimulus was accompanied by a beep, which allowed for a latency measurement. Responses were recorded with a digital voice recorder.

Accuracy and reaction times (RT) were obtained. The RT analysis was performed after excluding self‐corrected and incorrect responses. Using PRAAT, the RT for each trial was measured from the onset of the beep to the onset of the naming. The difference between incongruent and neutral conditions for both accuracy and RT was referred to as the Stroop difference (Scott & Wilshire, 2010). To account for overall speed differences in responses (Faroqi‐Shah et al., 2018; Patra et al., 2020a, 2020b), we calculated the percentage Stroop ratio (%), which was calculated by dividing the Stroop difference (mean incongruent – mean neutral) by the mean of neutral and incongruent trials and then multiplying by 100. A smaller Stroop difference and percentage Stroop ratio indicates better inhibitory control:

$$\mathrm{Stroop}\;\mathrm{Difference}=RT_{INCONGRUENT\;TRIAL}-RT_{NEUTRAL\;TRIAL}$$

$$\mathrm{Percentage}\;\mathrm{Stroop}\;\mathrm{ratio}\;\left(\%\right)=\left[\frac{\frac{RT_{INCONGRUENT\;TRIAL}-RT_{NEUTRAL\;TRIAL}}{RT_{INCONGRUENT\;TRIAL}+RT_{NEUTRAL\;TRIAL}}}{2}\right]\times 100$$

Shifting between task‐sets (Trail Making Test). The test was used to assess mental set shifting (TMT; Reitan, 1986). It is one of the most widely used neuropsychological tests for assessing mental set shifting (Sánchez‐Cubillo et al., 2009). The test consists of two parts, A and B. In part A, participants were asked to connect 25 circled numbers (e.g., 1, 2, 3) distributed on a paper using a pen/pencil. In part B, participants were asked to connect the circles but alternating between circled numbers and letters (e.g., 1, A, 2, B, 3, C). Participants completed both parts of the test. Total time (s) was measured for parts A and B, therefore, achieving two scores: TMT‐A, TMT‐B. The dependent variables were the difference in time between TMT‐A and TMT‐B scores: B – A, which has been shown to be the best indicator of task switching ability of the TMT Sánchez‐Cubillo et al., 2009), and the ratio of TMT‐B/TMT‐A, which has shown to eliminate the influence of perceptual speed to some extent (Salthouse, 2011).

#### Auditory working memory (auditory backward digit span)

Memory span is the longest list of items that a person can repeat back in correct order immediately after presentation on 50% of all trials. Items may include words, numbers, or letters. The task is known as digit span when numbers are used. The Wechsler's Memory Scale—3 (Wechsler, 1997) was used to measure the backward recall of digit sequences. In this test, participants were verbally presented with an increasingly longer series of digits from two to nine with a rate of presentation of one digit per s. Testing ceased when the subject failed to accurately recall two consecutive trials at any one span size or when the maximal list length was reached (7 digits). Participants were asked to recall the digits in the reverse order. Backward span is thought to tap into working memory (Wilde et al., 2004).

Table 2 provides the performance of each PWA across the executive control measures along with the mean performance from the HC group. The two groups differed significantly on all executive control measures (see Table 2 for the statistical findings). PWA demonstrated: a larger percentage Stroop ratio, which is indicative of poorer inhibitory control; a larger TMT ratio indicative of lesser shifting ability and flexibility; and a shorter span for working memory. Statistical analysis of individual PWA data using methods described by Crawford and Howell (1998) and Crawford and Garthwaite (2002) (detailed below) highlights the following: first, not all participants were consistently impaired on all three domains of executive control measures (e.g., P3Br2, P4Br2, P7Mx3 and P5An3 were only affected on memory span; P13Co4 was affected on shifting and memory span; P1An4, P2Co4 and P11Br2 were affected on all three domains; and P10An4 was affected on inhibitory control and shifting). This variability in performance at the individual level shows that having difficulty in one executive control component does not imply impairment across multiple domains, and executive control impairment at a group level does not reveal the complete picture (some individuals performed similarly to controls). Second, in comparison with the control data, PWA were most affected in the memory span domain (64% of the population, 9/14), whereas flexibility and shifting abilities were the least affected domain (43% of the population, 6/14) and half of the population (58% of the PWA population, 7/12) showed poor inhibitory control abilities. Third, to highlight is the usefulness of using ratio measures to capture the speed differences for PWA. Being slower in completing a task does not necessarily mean poorer executive control and vice versa (cf. the Stroop interference measures of P5An3and P14Br4, respectively).

**TABLE 2:** Performance of each person with aphasia (PWA) across the executive control measures, mean and SD of PWA and healthy controls (HC) groups, and statistical results comparing the groups

**PWA**	**Aphasia type** **^a^**	**Aphasia severity** **^a^**	**Stroop neutral RT (s)**	**Stroop incongruent RT (s)**	**Stroop difference**	**Stroop ratio**	**TMT Part A (s)**	**TMT Part B (s)**	**TMT difference**	**TMT ratio**	**Backward digit span**
P1An4	Anomic	4	703.3	2053.8	1350.6	98.0	80	356	276	4.5	2
P2Co4	Conduction	4	1963.4	3775.7	1812.3	63.2	56	225	169	4.0	2
P3Br2	Broca's	2	2656.1	3499.2	843.2	27.4	119	282	163	2.4	2
P4Br2	Broca's	2	2542.4	3026.6	484.3	17.4	80	154	74	1.9	2
P5An3	Anomic	3	965.0	1294.0	329.0	29.1	43	110	67	2.6	3
P6Co2	Conduction	2					73	184	111	2.5	4
P7Mx3	Mixed	3	1680.9	2625.1	944.2	43.9	33	105	72	3.2	3
P8Tc3	TCM	3	580.0	2008.2	1428.2	110.4	47	124	77	2.6	4
P9An4	Anomic	4					53	73	20	1.4	6
P10An4	Anomic	4	1078.6	4392.4	3313.8	121.1	43	151	108	3.5	4
P11Br2	Broca's	2	1628.3	5276.9	3648.7	105.7	59	227	168	3.9	0
P12Br4	Broca's	4	664.7	3472.3	2807.6	135.7	196	388	192	2.0	5
P13Co4	Conduction	4	2507.0	3314.0	807.0	27.7	51	201	150	3.9	2
P14Br4	Broca's	4	607.0	1077.0	470.0	55.8	40	97	57	2.4	2
Mean (PWA)	3.2	1464.7	2984.6	1519.9	69.6	69.5	191.2	121.7	2.9	2.9	
SD (PWA)	0.9	804.9	1240.3	1145.0	42.2	42.7	96.5	68.1	0.9	1.5	
Mean (HC)		662.0	888.1	226.1	28.5	29.8	63.8	34.0	2.1	5.4	
SD (HC)		138.2	212.5	147.8	15.0	10.1	37.4	29.9	0.6	1.1	
Statistical difference between groups. Effects sizes *d* > 1 for all tests					*U* ^b^ = 8.5, *p* < 0.05	*U* ^b^ = 53.0, *p* < 0.05			*U* ^b^ = 27.5, *p* < 0.05	*U* ^b^ = 78.5, *p* < 0.05	*U* ^b^ = 34.0, *p* < 0.05

*Note*: ^a^Type and severity of aphasia were classified based on Boston Diagnostic Aphasia Examination (BDAE) (Goodglass, Kaplan & Barresi, 2001).

^b^ Mann–Whitney *U*‐test; Crawford and Howell's (1998) statistical test was used to compare each PWA's score with the HC group. The Singlism.exe program (2002) was used to compute the statistics and text in shaded cells represent a significant difference (*p* < 0.05) between an individual PWA's score compared with the HC group mean.

### Analysis

For every participant, all variables were measured for each trial for the two fluency conditions. To arrive at a mean score for each variable for the letter fluency condition, three trials (F, A, S) were averaged. We implemented inferential statistics on the following variables: number of CR, FDS, 1st‐RT, Sub‐RT, cluster size, number of switches, within‐cluster pause and between‐cluster pause. As mentioned in the Participants section, we tested 14 PWA and 24 HC. It is notoriously difficult to establish well‐matched groups of PWA that are large enough to yield sufficient statistical power. Using G*Power (Faul et al., 2007, 2009), we established that with our participant groups, we have just about enough statistical power to establish between‐group differences for large effects (with *d* = 0.8): For *α* = 0.05 and one‐sided tests, 1 – *β* = 0.75, with recommended minimal power being 0.8 (Cohen, 1988), which we obtain for slightly larger effects (*d* ≥ 0.85). Regarding within‐participant differences between semantic and letter fluency, effects sizes of *d* > 0.7 and ≥ 0.55 yield enough statistical power for PWA and HC, respectively. Independent samples *t*‐test were performed to establish group differences (PWA versus HC); dependent sample *t*‐tests were performed to establish condition differences (semantic versus letter). We report the effect sizes from these analyses along with exact *p*‐values, to indicate the robustness of the effects, which remain at *p* < 0.05 when we correct for multiple comparisons on a variable‐by‐variable basis (α*3).

For correlations, in the group of PWA (*n* = 14) effect size *r* needs to be ≥ 0.58 to obtain sufficient power (1 – *β* = 0.8) at *α* = 0.05; for HC (*n* = 24), *r* needs to be 0.47 at *α* = 0.05. To examine the relationship between the executive control measures and the verbal fluency variables, Spearman's correlations were performed separately for each group and each condition (semantic, letter). Although our sample size is in line with clinical studies, to be cautious in our interpretation to avoid possible false‐positives, we interpret correlations only when their effect sizes are large enough to warrant sufficient statistical power.

To facilitate understanding of individual variation and to capture the heterogeneity of aphasia data, raw scores from the fluency variables for each of the 14 PWAs were reported. Following Crawford and colleagues’ statistical method of comparing a single neuropsychological client's performance with a control group performance, we used significance testing by comparing each PWA's score to the mean score obtained in the control sample for that specific variable (one‐tailed significance testing; Crawford & Howell, 1998; effect sizes and confidence interval; Crawford & Garthwaite, 2002; Crawford et al., 2010). This allowed us to test whether an individual's score on a variable was significantly different from the score obtained in the control sample. We believe this comprehensive approach of reporting findings from both group and individual levels improves our understanding of impairments in aphasia and allows for a more nuanced approach of our interpretation of the literature.

## RESULTS

**FIGURE 1:**
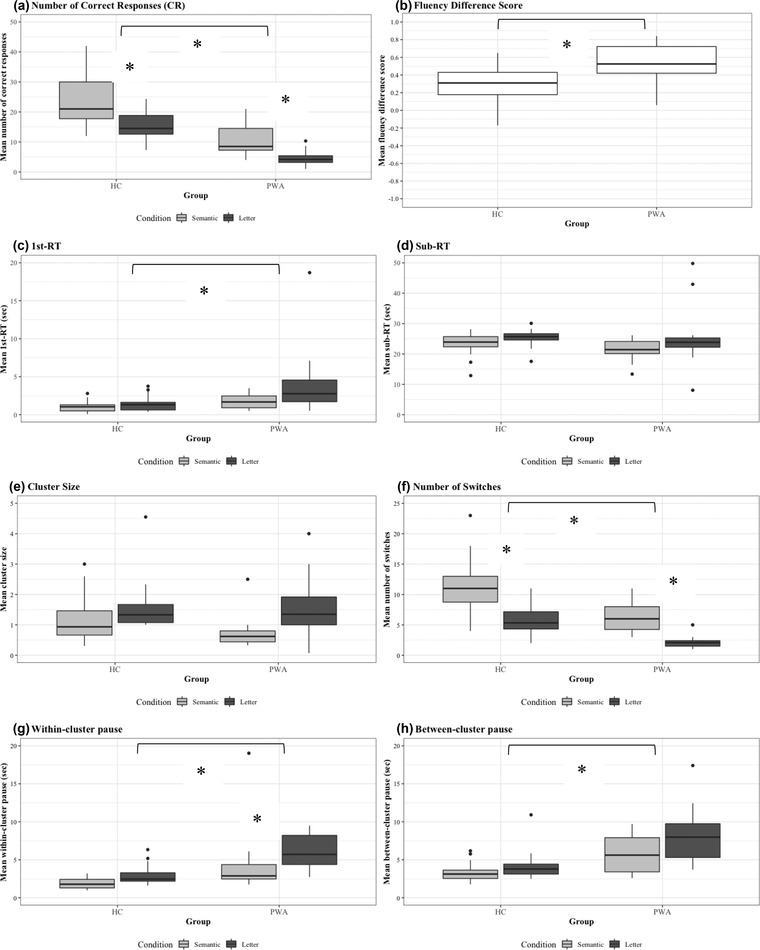
Box plots for the verbal fluency variables: (a) number of correct responses (CR); (b) fluency difference score (FDS); (c) 1st‐RT; (*d*) subsequent RT; (e) cluster size; (f) number of switches; (g) within‐cluster pauses; and (h) between‐cluster pauses *Note*: Lower and upper box boundaries are the 25th and 75th percentiles, respectively. A line inside a box represents the median. Lower and upper error lines are the 10th and 90th percentiles, respectively. Filled circles represent data falling outside the 10th and 90th percentiles. *Significant difference (between‐group differences for effect sizes of *d* ≥ 0.85; within‐participant differences between semantic and letter fluency, effects sizes of *d* > 0.7 and ≥ 0.55 for PWA and HC, respectively

Figure 1 presents the performance of the two groups (PWA, HC) for the verbal fluency variables as a function of condition (semantic, letter). The means and standard deviations (SD) for the verbal fluency variables for group (PWA, HC) and condition (semantic, letter) averaged across participants (SD reflects between‐subject variation), along with the results of the statistical analyses are presented in Appendix B. Individual PWA data for the fluency variables for semantic and letter conditions, along with the results of single‐subject Crawford statistics, are presented in Table 3. Findings from the correlation analyses between the executive control measures and the verbal fluency variables for each group are presented in Table 4. Interested readers are welcome to contact the first author if they wish to explore or interrogate anonymised item‐level verbal fluency time stamped data to test their research questions or to integrate these data in their own research.

**TABLE 3:** Raw score of each person with aphasia (PWA) in semantic and letter fluency conditions for all the verbal fluency variables (except FDS), and mean and SD of PWA and healthy controls (HC) groups

**PWA**	**Aphasia type**	**CR**			**1st‐RT**		**Sub‐RT**		**Cluster size**		**Number of switches**		**WCP**		**BCP**	
		**Semantic**	**Letter**	**FDS**	**Semantic**	**Letter**	**Semantic**	**Letter**	**Semantic**	**Letter**	**Semantic**	**Letter**	**Semantic**	**Letter**	**Semantic**	**Letter**
P1An4	Anomic	17	3.67	0.78	1.35	3.83	24.44	22.14	0.5	2	11	1.5	2.7	5.29	3.39	7.9
P2Co4	Conduction	6	5.67	0.06	2.4	2.42	23	26.19	0.6		4		19.03	5.48	9.7	8.57
P3Br2	Broca's	7	2.5	0.64	3.5	4.5	16.45	42.95	0.4	1	4	1.5	1.75	8.43	8	12.45
P4Br2	Broca's	8	4.67	0.42	1.75	7.11	19.99	25.5	0.44	3	8	1.5	2.89	8.86	7.65	8.06
P5An3	Anomic	8	4.67	0.42	0.89	1.48	21.4	19.14	0.33	0.6	6	2.5	1.79	9.49	8.77	7.19
P6Co2	Conduction	13	3	0.77	0.5	4.78	24.57	24.16	0.64	4	10		2.43	7.58	2.59	17.41
P7Mx3	Mixed	9	4.33	0.52	2.99	2.86	19.63	24.59	0.86	1	6	1.5	2.59	2.75	5.25	11.33
P8Tc3	TCM	6	4	0.33	2.5	2.42	23.42	18.84	0.75	0.07	3	1	6.1	4.3	9.67	10.14
P9An4	Anomic	21	10.33	0.51	0.99	0.51	26.16	22.51	2.5	1.56	5		3.66	3.44	2.83	3.71
P10An4	Anomic	10	8.67	0.13					0.67	2.5	5	3				
P11Br2	Broca's	4	1	0.75	1.6	18.7	13.37	49.8	0.4	1	4	3	5.9	4.6	3.48	5.24
P12Br4	Broca's	17	8	0.53	0.55	1.03	21.45	23.53	0.82	1.14	10	5	2.88	2.73	3.3	5.14
P13Co4	Conduction	8	3.67	0.54	0.55	1.27	24.35	22.38	0.44	1.67	8	2	2.22	8.48	6.5	5.46
P14Br4	Broca's	15	2.33	0.84	1.95	2.67	20.83	8.06	1	1	7	1	3.08		5.05	4.61
Mean (PWA)		10.64	4.75	0.52	1.66	4.12	21.47	25.37	0.74	1.58	6.5	2.14	4.39	5.95	5.86	8.25
SD (PWA)		5.09	2.62	0.24	0.98	4.74	3.56	10.49	0.54	1.07	2.56	1.19	4.61	2.49	2.66	3.83
Mean (HC)		23.83	15.71	0.31	1.01	1.4	23.58	25.46	1.15	1.5	11.08	5.82	1.87	2.91	3.3	4.08
SD (HC)		7.83	4.74	0.2	0.7	0.92	3.66	2.43	0.69	0.73	4.27	2.18	0.69	1.26	1.17	1.68

*Note*: Crawford and Howell's (1998) statistical test was used to compare each PWA's score with the HC group. The Singlism.exe program (2002) was used to compute the statistics and text in shaded cells represent a significant difference (*p* < 0.05) between an individual PWA's score compared with the HC group mean.

**TABLE 4:** Correlation coefficients between the executive control measures and the verbal fluency measures for each condition (semantic, letter) and group (PWA, HC)

**Executive control variables**		**Semantic fluency condition**						
		**CR**	**1st‐RT**	**Sub‐RT**	**Cluster size**	**Switches**	**WCP**	**BCP**
		PWA (*N* = 14)						
Stroop ratio	*rs* ^a^	0.208	–0.182	0.282	0.453	–0.110	0.564	–0.355
TMT ratio	*rs* ^a^	–0.347	0.077	0.055	–0.295	–0.064	0.082	0.275
Backward digit span	*rs* ^a^	0.540	–0.318	0.545	0.601	0.077	–0.026	–0.322
		HC (*N* = 24)						
Stroop ratio	*rs* ^a^	–0.168	0.236	0.002	–0.105	–0.067	0.003	0.352
TMT ratio	*rs* ^a^	–0.330	–0.255	–0.188	–0.088	–0.031	0.322	0.445
Backward digit span	*rs* ^a^	0.136	–0.069	0.293	0.140	0.012	–0.174	–0.261
		**Letter fluency condition**						
		PWA (*N* = 14)						
Stroop ratio	*rs* ^a^	0.288	–0.260	–0.182	–0.088	0.365	–0.794	–0.327
TMT ratio	*rs* ^a^	–0.225	0.138	–0.011	–0.031	0.108	0.049	0.220
Backward digit span	*rs* ^a^	0.657	–0.595	–0.357	0.082	0.204	–0.466	–0.026
		HC (*N* = 24)						
Stroop ratio	*rs* ^a^	–0.244	0.253	0.375	0.340	–0.106	0.242	0.202
TMT ratio	*rs* ^a^	–0.233	–0.464	–0.229	–0.600	–0.035	0.001	0.131
Backward digit span	*rs* ^a^	0.180	–0.329	0.103	0.207	0.273	–0.322	–0.222

*Note*: ^a^Spearman's correlation; PWA, people with aphasia; HC, healthy controls; text in shaded cells represent significant correlations. For significant correlations, in the group of PWA (*n* = 14) effect size *r* needs to be ≥ 0.58 to obtain sufficient power (1 – *β* = 0.8) at *α* = 0.05, for healthy controls (*n* = 24), *r* needs to be 0.47, both for *α* = 0.05.

### Group and condition differences in verbal fluency performance

Significant differences between PWA and HC were observed for CR, FDS, 1st‐RT, number of switches, within‐cluster pauses and between‐cluster pauses. There were no group differences in Sub‐RT and cluster size. There were several main effects of condition in within participant analyses. See Figure 1 and Appendix B for the results and the statistical tests.

The CR showed a significant effect of group (PWA: mean = 7.70, SD = 3.42; HC: mean = 19.77, SD = 5.55) and a significant effect of condition for both PWA and HC in within participant analyses (PWA_semantic_: mean = 10.64 SD = 5.09; PWA_letter_: mean = 4.75, SD = 2.62; HC_semantic_: mean = 23.83, SD = 7.83; HC_letter_: mean = 15.71, SD = 4.73). PWA produced fewer exemplars during the fluency task; both groups produced fewer CR for the letter than the semantic condition. For FDS, there was a significant effect of group (PWA: mean = 0.52, SD = 0.24; HC: mean = 0.31, SD = 0.2). A significantly larger FDS score for PWA indicates difficulty in maintaining performance in the difficult letter fluency condition; this is indicative of poorer executive control abilities.

In terms of timing, the interpretation of 1st‐RT and Sub‐RT need to be made in conjunction. The 1st‐RT is the initial speed to access the mental lexicon; whilst Sub‐RT indicates the recall rate and the time point where half of the responses have been generated. The 1st‐RT showed a significant effect of group (PWA: mean = 2.89, SD = 2.47; HC: mean = 1.21, SD = 0.62) and no effects for within participant analyses for either PWA or HC. A slower 1st‐RT alongside parallel Sub‐RT times suggests that PWA took longer than HC to access their lexical store when retrieving the first exemplar and that PWA did not generate exemplars for as long as HC did (otherwise Sub‐RT would be shifted, too). Comparable Sub‐RTs across groups highlight that the decline in the rate of recall was similar across groups, despite the fact that PWA had fewer number of exemplars.

In the context of clustering and switching analyses, there were significant effects of group for number of switches (PWA: mean = 4.77, SD = 2.03; HC: mean = 8.45, SD = 2.82), within‐cluster pause (PWA: mean = 5.05, SD = 2.43; HC: mean = 2.42, SD = 0.73), and between‐cluster pause (PWA: mean = 7.05, SD = 2.45; HC: mean = 3.72, SD = 1.20). For PWA there were significant condition effects for number of switches (semantic: mean = 6.54, SD = 2.54; letter: mean = 2.14, SD = 1.18). For HC, there were significant condition effects for number of switches (semantic: mean = 11.08, SD = 4.27; letter: mean = 5.82, SD = 2.18) and within‐cluster pause (semantic: mean = 1.86, SD = 0.69; letter: mean = 2.88, SD = 1.28).

These results suggest that compared with HC, PWA switched fewer number of times, and took longer to access words within clusters and to switch between clusters. Importantly, there was not a main effect of group for cluster size, which suggests that once PWA were able to access subcategories they were able to generate a similar number of words within those clusters, albeit more slowly. Similar clustering strategies but difficulty in switching was indicative of difficulty of executive control abilities.

Table 3 presents the raw scores of each PWA in each condition for the verbal fluency variables. At the individual level, we observed letter fluency to be more difficult compared with semantic fluency across all the variables (number of CR, cluster size, number of switches, pause durations). For example, for number of CR, 9/14 PWA (64%) were affected on semantic fluency, but 11/14 PWA (79%) showed a significantly lower score on letter fluency. Similarly, number of switches for PWA were affected more in letter condition (7/11 PWA, 64%) compared with semantic condition (4/14 PWA, 29%).

### Verbal fluency performance and executive control measures

Table 4 presents the correlation coefficients amongst the verbal fluency variables for letter and semantic fluency conditions and executive control measures for PWA and HC.^1^ The only significant correlation for the semantic fluency and executive control measures was a positive correlation between backward digit span and cluster size for PWA. In contrast, for the letter fluency condition, PWA showed significant correlations between backward digit span and number of correct responses (positive), backward digit span and 1st‐RT (negative), and between Stroop ratio and within‐cluster pauses (negative). The HC showed only a significant correlation between TMT ratio and cluster size (negative). These findings indicate that PWA with better working memory were able to produce more correct responses and access those responses faster on the difficult letter fluency condition, and also had larger cluster sizes in semantic fluency condition. Further, PWA with better inhibitory control took longer time to search within a cluster. This could be attributed to the better inhibitory control abilities by which those PWA could avoid interference from other subclusters. In addition, increased shifting and flexibility in HC was associated with producing larger cluster sizes.

## DISCUSSION

Using the multidimensional analysis approach for verbal fluency performance from both semantic and letter fluency trials, this is the first study to systematically research if deficient verbal fluency performance in PWA was due to lexical and/or executive control difficulties. Specifically, we aimed at (1) characterizing differences between PWA and HC across a range of variables characterizing both semantic fluency and letter fluency performance, and at (2) establishing the relationship between verbal fluency performance and executive control in PWA and HC. The findings are organized in Table 5 with respect to the group differences (PWA versus HC), effect of condition (semantic versus letter), and relationship of verbal fluency variables with the executive control measures. The picture that emerges from Table 5 is that at a group level, PWA have significant difficulties in *both* lexical and executive control component of the verbal fluency task. The variables that depended on lexical processes were impaired, such as, CR, 1st‐RT, within‐cluster pauses; so were variables that depended on the executive control processes, such as FDS, number of switches, between‐cluster pauses. It is important to also note that PWA were similar to controls in cluster size and Sub‐RT. At the individual level, PWA had greater difficulty on the letter fluency condition compared with semantic fluency condition. Correlational findings provided further support for this as significant correlations between the executive control and verbal fluency measures were mostly observed for the letter fluency condition. It is of note, however, that contrary to what one might expect, the correlations we observed were not with those fluency measures that are thought to rely strongly on executive functions but with those measures that are thought to reflect the lexical aspect of the fluency task or both the lexical and the executive aspects. This might suggest that the correlations we observed reflect the interplay of executive and lexical processes in word retrieval rather than word retrieval per se. We will discuss the findings summarized in Table 5 in the context of the literature to better understand the organization mental lexicon following aphasia, and to elucidate the mechanisms that they might be implementing to perform these fluency tasks.

**TABLE 5:** Results of the present study in the context of the verbal fluency variables and their lexical and executive control components

**Parameters**	**Lexical**	**Executive**	**People with aphasia (PWA) versus healthy controls (HC)**		
	**processes**	**control processes**	**Group effect**	**Condition effect**	**Correlation with executive control**
*Quantitative analysis*					
Number of correct responses (CR)	✓	✓	Yes, PWA < HC. PWA fewer exemplars in both semantic and letter conditions	Semantic > letter (yes, both groups)	Yes, for letter fluency, (+) with backward digit span for PWA
Fluency difference score (FDS)^a^		✓	Yes, PWA > HC. PWA higher FDS score	Not applicable	
*Time‐course analysis* ^b,c^					
1st–RT	✓		Yes, PWA > HC. PWA longer 1st‐RT, significantly longer for letter	Semantic = letter (both groups)	Yes, for letter fluency, (–) with backward digit span for PWA
Sub‐RT		✓	No, PWA = HC	Semantic = letter (both groups)	
*Clustering and switching analysis* *^d^*					
Cluster size	✓		No, PWA = HC	Semantic = letter (both groups)	Yes, for semantic fluency, (+) with backward digit span for PWA; Yes, for letter fluency, (–) with TMT ratio for HC
Number of switches		✓	Yes, PWA < HC. PWA switched fewer times than HC	Semantic > letter (Yes, both groups)	
Within‐cluster pauses	✓		Yes, PWA > HC. PWA had longer within‐cluster pauses	Semantic > letter (only for HC)	Yes, for letter fluency, (–) with Stroop ratio for PWA
Between‐cluster pauses		✓	Yes, PWA > HC. PWA had longer between‐cluster pauses	Semantic = letter (both groups)	

Sources: ^a^Friesen et al. (2015); ^b^Luo et al. (2010); ^c^Sandoval et al. (2010); ^d^Troyer et al. (1997). Adapted from Patra et al. (2020a, 2020b).

### Effects of group and condition on verbal fluency performance

PWA retrieved and generated fewer correct words for both types of fluency trials; this is in concurrence with the aphasia literature which has consistently shown PWA to have difficulties in lexical retrieval and production (Adams et al., 1989; Arroyo‐Anlló et al., 2012; Baldo et al., 2010; Bose et al., 2017; Faroqi‐Shah & Milman, 2018; Kiran et al., 2014; Patra et al., 2020b; Roberts & Dorze, 1994; Sarno et al., 2005). Individual level performance indicates that letter fluency is more difficult compared with semantic fluency across all the variables for PWA (number of CR, cluster size, number of switches, pause durations). This could be due to PWAs’ difficulty with the executive component, as indicated by larger FDS values, compared with HC. FDS measures the ability to maintain performance in the demanding letter fluency condition, capturing the executive component of the verbal fluency task. Individuals who can maintain better performance in the difficult letter fluency condition would show a smaller FDS score; this has been taken to be indicative of better executive control abilities (Friesen et al., 2015; Patra et al., 2020a). PWA showed larger FDS scores indicating that they had difficulty in the executive component of the task and in maintaining performance in the letter fluency trials.

The timing measures revealed that: PWA were slower than HC in getting started in both types of trials as measured by 1st‐RT; difficulty with the speed in retrieving of the words was also evident in significantly longer within‐ and between‐cluster pauses. Difficulty in retrieval speed during verbal fluency corroborates previous studies which have shown increased pause durations for PWA during fluency tasks (Bose et al., 2017; Patra et al., 2020a). The finding of higher 1st‐RT with lower number of CR, indicate that they took longer to retrieve the first word of their response and retrieved fewer words overall. In contrast, the Sub‐RT, that is, the average time elapsing between the onset of the first response and the onsets of each later word in the response, was similar across groups. This suggests that PWA probably stopped generating words earlier than healthy adults did, which would be compatible with the finding that they had a lower number of CR. Critically, however, the decline in recall from the first to the last exemplar generated by the participants, reflected in Sub‐RT, was comparable between groups. This decline in the rate of recall is taken to be an indicator of the structural integrity of the mental lexicon (Rohrer et al., 1995). Therefore, taken together, we would interpret our findings as indicating a retrieval problem rather than a structural lexical problem being the cause of the poorer performance of PWA as compared with controls.

The finding of equivalent cluster sizes for PWA and HC was surprising given that past research has shown PWA usually have smaller cluster sizes (Baldo et al., 2010; Bose et al., 2017; Kiran et al., 2014; Sarno et al., 2005). Clustering involves accessing and using the word store; cluster size is a measure of the ability to access words within semantic subcategories. Our finding is in contrast to most of the research showing smaller cluster size in PWA, which included only semantic fluency. Analysis of cluster size for individual PWA shows only four instances out of a possible 28 (once in semantic, thrice in letter) was cluster size significantly different from controls (Table 3). It has been proposed that cluster size is a good indication of the integrity of the semantic store (Kavé et al., 2011; Troyer, 2000; Velázquez‐Cardoso et al., 2014). Based on the group and individual participant analyses, similar cluster size in the PWA would imply that when they were able to access the subcategory they were able access similar number of words compared with the controls within that subcategory, albeit slowly as indicated by slower within‐cluster pauses.

Switching involves the search processes and is a measure of the ability to shift efficiently from one subcategory to another; reduced switching has been attributed to an executive function difficulty to shift between subcategories (Troyer et al., 1997). Several previous studies have shown that switching is a strong predictor for total correct in both typical and clinical populations, such as aphasia (e.g., Bose et al., 2017; Faroqi‐Shah & Milman, 2018; Kiran et al., 2014; Troyer et al., 1997). The finding of fewer number of switches with increased between‐cluster retrieval times further reveals the involvement of effortful and controlled retrieval processes from the word store (e.g., Bose et al., 2017; Gruenewald & Lockhead, 1980; Rosen et al., 2005). PWA experienced greater difficulty with effective search strategies for subcategories highlighting the possible difficulties with the executive component of the task. This once again demonstrates that as the search gets more effortful, executive control components are stretched.

### Executive control measures across groups and fluency conditions

Difficulty in the executive control components of the verbal fluency task for the PWA was further supported by the results obtained from the separate executive control measures. As expected, compared with controls, PWA showed significantly larger Stroop ratio, which is indicative of a difficulty with inhibitory control. The findings are consistent with previous studies on aphasia and inhibitory control (Faroqi‐Shah et al., 2018; Patra et al., 2020a). On the task switching measure, PWA showed a larger TMT ratio compared with control; this is indicative of a difficulty with switching between mental sets. Previous studies have shown PWA to have difficulty in task switching compared with healthy adults (Helm‐Estabrooks, 2002). In terms of memory spans, previous studies have shown difference in memory spans between PWA and control participants (see Murray et al., 2018, for a review). Importantly, individual level analysis revealed that not all the PWA showed executive control impairments on all the domains. A larger portion of PWA were affected on inhibitory control (about 56%), flexibility and shifting (about 43%) and memory spans (about 64%). This data also highlights the importance of using ratio measures for variables that depend on the speed to compensate for general speed differences in neurological impairments. If we had solely depended on raw difference measures; then the proportion of PWA affected in Stroop and TMT were 92% and 57%, respectively. In contrast, it was 56% for Stroop and 43% for TMT when we considered ratio measures. This highlights the dangers of inflating the possibility of finding executive control differences between PWA and controls. These results also signify the importance of including a broad range of executive control measures; on importance of performing individual level data analysis for PWA; and the use of speed corrected measures.

Finally, the correlation analyses revealed associations mostly between letter fluency and executive control measures. It is important to reiterate that the correlations we observed were not with those fluency measures that are thought to rely strongly on executive functions but with those measures that are thought to reflect the lexical aspect of the fluency task (i.e., 1st‐RT, within cluster pause, cluster size) or both the lexical and the executive aspects (i.e., CR). PWA with larger spans produced a greater number of correct responses and were faster to access the lexicon in the letter fluency condition; larger spans resulted in larger cluster size in semantic fluency condition. Recall that memory spans were the domain where most of the PWAs were affected (9/14 people). Moreover, PWA who exhibited better inhibitory control took longer to search within a cluster. Within cluster pauses are thought to reflect search time within a subcategory. There could be two possible explanations for the finding of a positive correlation between better inhibitory control and within cluster pause duration. PWA with better inhibitory control may have taken longer to search within a cluster in order to avoid interfering responses from other subcategories, and hence avoid errors that PWA with less efficient inhibitory control may have produced. Alternatively, it is possible that PWA with better inhibitory control may have taken longer to search within a cluster in an attempt to find more exemplars within a cluster or had greater difficulty switching to other clusters that they had inhibited before. Note that there were no correlations between executive control measures and cluster size or between executive control measures and the number of switches, but there were between executive control measures and the number of CR. For the time being, these alternative explanations must remain as speculative interpretations of the findings because we have little means to corroborate them with our data.

We are hopeful that this detailed investigation will instigate further research to address some of the limitations and outstanding questions of the current research. Future studies with larger sample sizes and variable working memory deficits, or collation of data from existing published studies, could further inform how specific measures of executive control relate to verbal fluency performance. Specifically, future studies would benefit from using sensitive experimental measures of executive control with varying linguistic loads. In clinical populations with linguistic impairments, such as aphasia, linguistic load of executive control tasks could modulate the performance on these tasks. Another avenue that stands to inform the topic of semantic organisation of mental lexicon in aphasia would be to use different types of semantic trials, such as, comparison of large (e.g., animals, supermarket items) versus smaller semantic categories (e.g., vehicles, fruits). In the context of aphasia rehabilitation, it is possible that PWA who show deficits in both verbal fluency conditions would benefit from therapy targeting executive control in addition to traditional language therapy. However, this needs to be tested in future research.

## CONCLUSIONS

To the best of our knowledge, this is the first study to undertake a systematic and comprehensive analysis approach to explore the lexical and executive control processes underlying verbal fluency performance in PWA. The key findings were that the variables that depended on lexical processes were impaired, such as, CR, 1st‐RT, within‐cluster pauses; so were variables that depended on the executive control processes, such as FDS, number of switches, slope, between‐cluster pauses. Importantly, PWA were similar to control in cluster size and Sub‐RT. For PWA, Stroop ratio and backward digit spans were the only executive control measures that demonstrated any correlation with some of the letter fluency variables. In the present study, intact semantic comprehension on the background language task, similar clustering strategy, intact retrieval of words within a cluster with impaired switching, and longer between‐cluster pauses for the PWA indicate impaired lexical access and greater impairment in the executive control components of the verbal fluency tasks (Shao et al., 2014). Further, previous studies had speculated on the role of executive control in verbal fluency measures, but the present study used separate experimental measures of executive control to confirm that memory spans play a key role in the verbal fluency performance in PWA, especially in letter fluency. In summary, the results of this detailed multidimensional analysis approach for verbal fluency performance revealed that PWA had significant difficulties in *both* lexical and executive control components of the verbal fluency tasks. This research makes a significant contribution to our understanding of lexical and executive control aspects in word production in aphasia. From a clinical perspective, this research highlights the importance of using a full range of verbal fluency and executive control measures to tap into the lexical as well as executive control abilities of aphasia, and also the utility of using letter fluency to tap into the executive control processes in aphasia. Importantly, if one relies only on a semantic condition, we might not be able tease apart the impact of executive control processes.

## CONFLICT OF INTEREST

The authors declare no conflict of interest.

## NOTE

1 Following a recommendation by the reviewers, we performed correlational analyses between PNT scores and CR for semantic and letter trials for PWA. We observed a significant positive correlation between PNT accuracy and CR semantic trials (*r*_s_ = 0.64; *p* = 0.01), but no significant correlation between PNT scores and CR letter trials (*r*_s_ = 0.16; *p* = 0.59). Moreover, there were no significant correlation between PNT scores and executive control measures.

## APPENDIX A

**TABLE A1:** Demographic profiles of each person with aphasia (PWA), mean and SD of PWA and healthy controls (HC) groups, and the raw scores in the various background measures

**PWA**	**Age**	**Sex**	**YoE** **^a^**	**Time post‐onset (months)**	**Aphasia type** **^b^**	**Aphasia severity** **^b^**	**Right hemiparesis**	**PNT** **^c^** **(*N* = 175)**	**PNT** **^c^** **(%)**	**PPT‐3 Pictures Version** **d** **(*N* = 52)**	**PALPA #47: Spoken Word–Picture Matching (*N* = 40)** **^e^**	**Lexical comprehension (*N* = 16)** **^f^**	**PALPA #9: Word Repetition (*N* = 80)** **^e^**	**PALPA #8: Non‐word Repetition (*N* = 30)** **^e^**	**PALPA #2: Real‐word Minimal Pair Discrimination (*N* = 72)** **^e^**	**PALPA #4: Minimal Pair Requiring Picture Selection (*N* = 40)** **^e^**
P1An4	74	M	13	51	Anomic	4	No	145	83	96.2	97.5	100	97.5	73.33	91.66	95
P2Co4	51	M	15	22	Conduction	4	Yes, recovered	96	54.86	96.15	97.5	100	61.25	26.67	94.44	97.5
P3Br2	60	F	11	48	Broca's	2	Yes	130	74.28	92.31	97.5	100	83.75	30	91.67	97.5
P4Br2	60	M	11	115	Broca's	2	Yes	142	81.14	98	100	100	100	93.3	100	95
P5An3	63	F	14	84	Anomic	3	No	159	91	98	100		100	100	99	
P6Co2	71	M	20	192	Conduction	2	Right hemiparesis	136	77.7	98	98		91.3	56.6	94	98
P7Mx3	58	F	14	14	Mixed	3	Moderate R‐sided	159	91	94.23	100	100	92.5	83.33	98.6	100
P8Tc3	59	M	13	47	TCM	3	No	131	74.85	98	100	93.75	93.75	83.33	100	100
P9An4	70	F	14	48	Anomic	4	No	158	90.28							
P10An4	79	F	15	48	Anomic	4	No	131	74.85	90.4	97.5	100	95	100	97.22	97.5
P11Br2	77	M	13	56	Broca's	2	No	125	71.43	94.23	97.5	100	67.5	70	93.05	92.5
P12Br4	61	M	11	120	Broca's	4	No	145	82.86	96.15	100	93.75	97.8	66.67	95.83	97.5
P13Co4	52	F	13	145	Conduction	4	Mild R‐sided weakness	59	33.7	94.2			63.7	33.3		
P14Br4	51	F	16	48	Broca's	4	R‐sided weakness	168	96	96	100		100	87	100	
*Mean* (PWA)	63.3		13.8	74.1		3.2		134.6	76.9	95.5	98.8	98.6	88.0	69.5	96.3	97.1
*SD* (PWA)	9.4		2.4	50.7		0.9		28.4	16.2	2.4	1.3	2.8	14.4	25.8	3.3	2.3
*Mean* (HC)	67.33		14					167.19	95.53							
*SD* (HC)	10.09		3.06					5.13	2.98							

Note: ^a^Years of education.

^b^ Type and severity of aphasia were classified based on Boston Diagnostic Aphasia Examination (BDAE; Goodglass, Kaplan & Barresi, 2001).

^c^ Philadelphia Naming Test (PNT; Roach et al., 1996).

^d^ Pyramid and Palm Trees Test (PPT; Howard & Pattinson, 1992).

^e^ Psycholinguistic Assessments of Language Processing in Aphasia subtests (PALPA; Kay, Lesser, & Coltheart, 1992).

^f^ The Philadelphia Comprehension Battery (Saffran et al., 1987).

## APPENDIX B

**TABLE A2:** Statistical results of the verbal fluency variables by group (PWA, HC) and condition (semantic, letter)

	**HC (*N* = 24)**		**PWA (*N* = 14)**		**Total (*N* = 38)**		**Statistical results** **^a^**		
	**Mean**	**SD**	**Mean**	**SD**	**Mean**	**SD**	**Group difference (PWA versus HC)**	**PWA: condition difference (semantic versus letter)**	**HC: condition difference (semantic versus letter)**
CR^b^	19.77	5.55	7.70	3.42	13.73	4.48	*t*(36) = 7.34, *p* < 0.001, *d* = 2.62	*t*(13) = 5.08, *p* < 0.001, *d* = 1.36	*t*(23) = 5.98, *p* < 0.001, *d* = 1.22
*Semantic*	23.83	7.83	10.64	5.09	17.24	6.46			
*Letter*	15.71	4.73	4.75	2.62	10.23	3.68			
FDS^c^	0.31	0.20	0.52	0.24	0.41	0.22	*t*(36) = –2.92, *p* = 0.006, *d* = 0.95		
1st‐RT	1.21	0.62	2.89	2.47	2.05	1.54	*t*(35) = –3.19, *p* = 0.003, *d* = 0.93	*t*(12) = –1.88, *p* = 0.08, *d* = 0.52	*t*(22) = –1.75, *p* = 0.09, *d* = 0.36
*Semantic*	1.01	0.70	1.66	0.97	1.34	0.84			
*Letter*	1.40	0.94	4.12	4.74	2.76	2.84			
Sub‐RT	24.48	2.36	23.42	4.16	23.94	3.26	*t*(35) = 1.0, *p* = 0.32, *d* = 0.31	*t*(12) = –1.06, *p* = 0.31, *d* = 0.29	*t*(22) = –2.44, *p* = 0.02, *d* = 0.50
*Semantic*	23.58	3.66	21.47	3.56	22.53	3.61			
*Letter*	25.62	2.35	25.37	10.49	25.50	6.42			
Cluster size	1.33	0.48	1.12	0.59	1.23	0.53	*t*(36) = 1.16, *p* = 0.25, *d* = 0.39	*t*(12) = –2.43, *p* = 0.03, *d* = 0.67	*t*(23) = –1.63, *p* = 0.12, *d* = 0.33
*Semantic*	1.15	0.69	0.75	0.56	0.95	0.63			
*Letter*	1.50	0.73	1.58	1.07	1.54	0.90			
Number of switches	8.45	2.82	4.77	2.03	6.61	2.42	*t*(36) = 4.28, *p* < 0.001, *d* = 1.49	*t*(10) = 5.81, *p* < 0.001, *d* = 1.75	*t*(23) = 6.84, *p* < 0.001, *d* = 1.39
*Semantic*	11.08	4.27	6.54	2.54	8.81	3.41			
*Letter*	5.82	2.18	2.14	1.18	3.98	1.68			
WCP*^d^*	2.42	0.73	5.05	2.43	3.74	1.58	*t*(35) = –4.96, *p* < 0.001, *d* = 1.46	*t*(11) = –0.86, *p* = 0.41, *d* = 0.25	*t*(22) = –3.30, *p* = 0.003, *d* = 0.69
*Semantic*	1.86	0.69	4.49	4.79	3.18	2.74			
*Letter*	2.88	1.28	5.95	2.49	4.42	1.89			
BCP^e^	3.72	1.20	7.05	2.45	5.39	1.83	*t*(35) = –5.57, *p* < 0.001, *d* = 1.73	*t*(12) = –1.94, *p* = 0.08, *d* = 0.53	*t*(22) = –2.17, *p* = 0.04, *d* = 0.45
*Semantic*	3.30	1.17	5.86	2.66	4.58	1.92			
*Letter*	4.05	1.71	8.25	3.83	6.15	2.77			

*Note*: ^a^Independent samples *t*‐test were performed to establish group differences, and dependent sample *t*‐tests were performed to establish condition differences. PWA, people with aphasia; HC, healthy controls. Shaded text represents significant statistical results. Using G*Power (Faul et al., 2007, 2009), we established that with our participant groups we have just about enough statistical power to establish between‐group differences for effect sizes of *d* ≥ 0.85. Regarding within‐participant differences between semantic and letter fluency, effects sizes of *d* > 0.7 and ≥ 0.55 yield enough statistical power for PWA and HC, respectively.

^b^ Number of correct responses.

^c^ Fluency difference score.

^d^ Within‐cluster pause

^e^ Between‐cluster pause; condition (semantic, letter).
